# COVID‐19‐associated thrombotic angiopathy improved after plasma exchange

**DOI:** 10.1002/ccr3.4991

**Published:** 2021-11-25

**Authors:** Natalie Elkayam, Gagan Raju, Yiwu Huang, Jay Lipshitz, Stephen Peeke, Martin H. Bluth

**Affiliations:** ^1^ Department of Hematology and Oncology Maimonides Medical Center Brooklyn New York USA; ^2^ Department of Pathology Maimonides Medical Center Brooklyn New York USA

**Keywords:** angiopathy, coagulation, COVID, inflammation, plasma exchange, thrombosis, TTP

## Abstract

Utilization of therapeutic plasma exchange in select patients with COVID‐19 microangiopathy may provide useful treatment by modulation of inflammatory cytokines and coagulation cascade to maintain homeostasis.

## Graphical abstract

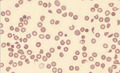

## 1 CASE PRESENTATION

Thrombotic angiopathy represents a dire consequence of COVID‐19 infection, and such endothelial cell dysfunction and microvascular thrombosis are thought to contribute to resultant multi‐organ complications. Treatments have included anti‐viral/anti‐bacterial agents, immunoglobulins, thrombolytics, and other immune modulators with variable results. Here, we present therapeutic plasma exchange (TPE) in such a patient with presumptive thrombotic thrombocytopenic purpura (TTP). A 44‐year‐old female patient received dexamethasone as standard of care for severe COVID‐19 infection and also received empiric TPE for presumptive TTP (polychromasia, basophilic stippling, schistocytes, and pending ADAMTS13). After plasma exchange, patient demonstrated significant improvement including decreases in lactate dehydrogenase (3802–945 IU/L)/total bilirubin (4.0–2.9 mg/dl)/schistocytes, increase in platelet count (18–68 ×10^9^/L), and declining vasopressor requirements. However, ADAMTS13 levels drawn prior to TPE was 50.7% decreasing probability of TTP diagnosis. COVID‐19‐associated thrombotic microangiopathy exhibited a dramatic response to TPE. It is conceivable that plasma exchange accounts for cytokine removal of inflammatory mediators in the plasma and reinstitution of immune homeostasis. Plasma exchange used on a limited scale, particularly for patients with COVID‐19 microangiopathy, may represent a useful treatment for a particularly devastating manifestation of COVID‐19.

A 44‐year‐old Chinese woman with no known medical history presented to the emergency department for evaluation of shortness of breath. The patient reported fevers, chills and a dry cough which had progressively worsened during the preceding week. Her husband was also sick at home with similar symptoms. Upon arrival to the emergency room, she was afebrile (36.4℃) with a blood pressure of 132/87 mm Hg, tachycardic (122 beats/minute) and tachypneic (25 breaths/minute). Her oxygen saturation was 80% on 6 L of oxygen via nasal cannula requiring escalation to a non‐rebreather mask on which her oxygen saturation declined to 75%. Due to progressive dyspnea and hypoxia refractory to oxygen supplementation, she was endotracheally intubated and mechanically ventilated on account of acute respiratory failure. Initial laboratory studies demonstrated creatinine of 1.0 mg/dl, lactate dehydrogenase (LDH) was 2334 IU/L and liver function tests (LFT) were aspartate transaminase (AST)/alanine transaminase (ALT) 79/63 IU/L with bilirubin levels within normal limits. Coronavirus‐2019 (COVID19) PCR was reactive. C‐reactive protein (CRP) was 38.6 mg/dl and ferritin were found to be 297.9 ng/ml. Her complete blood count (CBC) on presentation demonstrated the following: white blood cell (WBC) 13. × 10^9^cells/L, hemoglobin (Hgb) of 11.7 g/dl and platelet count of 243 × 10^9^/L. Coagulation profile including international normalized ratio (INR), activated partial thromboplastin time (aPTT) and fibrinogen were all in normal range. Patient was admitted to the medical intensive care unit for management of septic shock and acute respiratory failure secondary to acute COVID19 infection. She underwent CT angiography of the chest, which demonstrated no pulmonary embolism and bilateral, predominantly ground glass opacities consistent with COVID19 infection changes.

The patient received dexamethasone therapy as indicated in the standard of care for management of severe COVID19 infection. Within one day of her hospitalization, her laboratory findings demonstrated worsening renal function (blood urea nitrogen (BUN)/creatinine 45/2.2 mg/dl) and worsening total bilirubin up to 4.0 mg/dl within 3 days of her admission. The patient underwent continuous venovenous hemodiafiltration (CVVHD) for acute renal failure. She received prophylactic apixaban 2.5 mg Q12 per hospital COVID‐19 prophylactic anticoagulation protocol for one day and subsequently enoxaparin 40 mg daily on hospital days 2–3. Her troponin trended up to 1.41 ng/ml, which was attributed to demand ischemia due to underlying infectious process. Her WBC count remained stable throughout her admission. Her hemoglobin decreased to 6.8 g/dl and she received red blood cell (RBC) transfusion. Her platelet count within one day of admission dropped from 243 to 65 × 10^9^/L and further decreased to 23 × 10^9^/L, at which point her anticoagulation regimen was withheld for thrombocytopenia. Poly Coombs test was negative. Heparin induced thrombocytopenia (HIT) antibody was negative. Fibrinogen levels were not determined upon presentation but were assessed ten days after admission to the hospital and were found to be 385 mg/dl. Fibrinogen levels remained within the reference range (217–521 mg/dl) throughout the hospital course.

**FIGURE 1:**
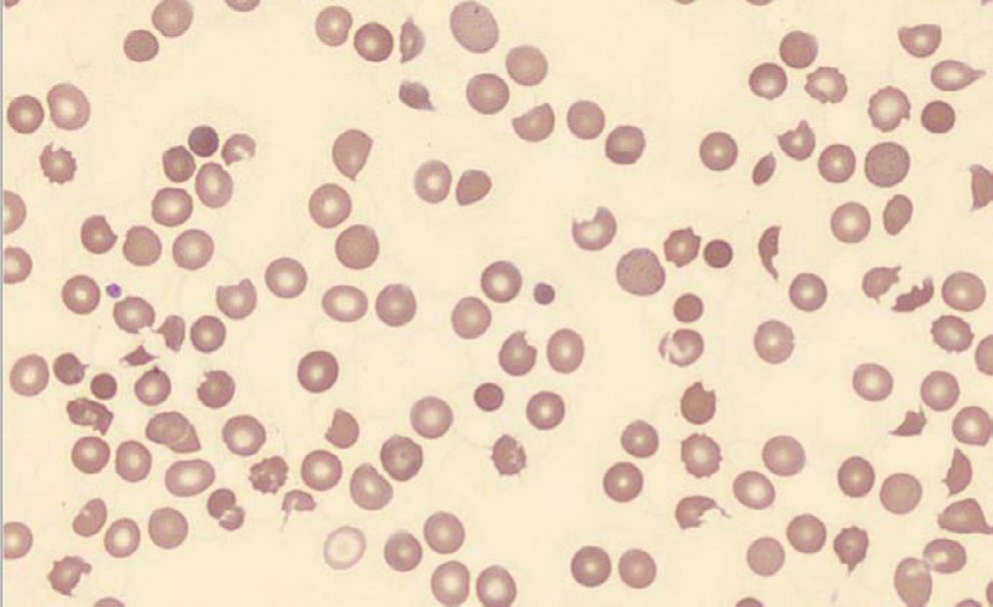
Peripheral smear with numerous observed schistocytes

Due to dropping hemoglobin and platelet values, hematology evaluation was obtained for assessment of possible thrombotic thrombocytopenic purpura (TTP). Peripheral smear demonstrated normochromic RBC with some polychromasia, numerous nucleated RBCs, occasional basophilic stippling with many schistocytes identified (approximately 5‐6/high power field) (Figure 1). No platelet clumps were identified though some large platelets were observed and manual platelet count estimated around 30–50 × 10^9^/L. Polymorphonuclear cells with toxic granules, many band cells and some large activated lymphocytes were identified. Blood samples were sent out for ADAMTS13 levels.

On account of the high clinical suspicion for thrombotic angiopathy, a decision was made to commence empiric plasma exchange in advance of receipt of ADAMTS13 level. The patient underwent plasma exchange, on day 4 of admission, with one blood volume of fresh frozen plasma (FFP) for five consecutive days.

**FIGURE 2:**
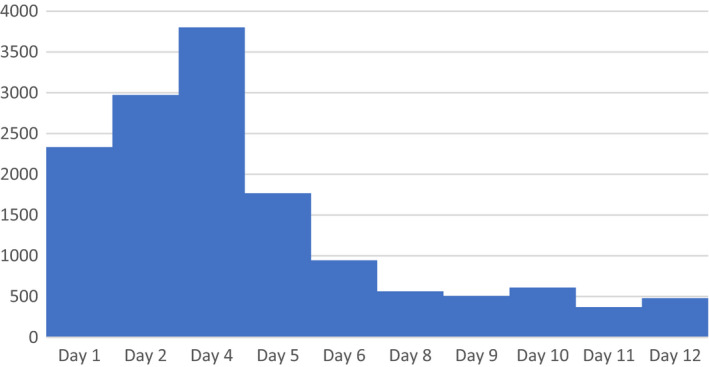
LDH levels during course of treatment (in IU/L)

**FIGURE 3:**
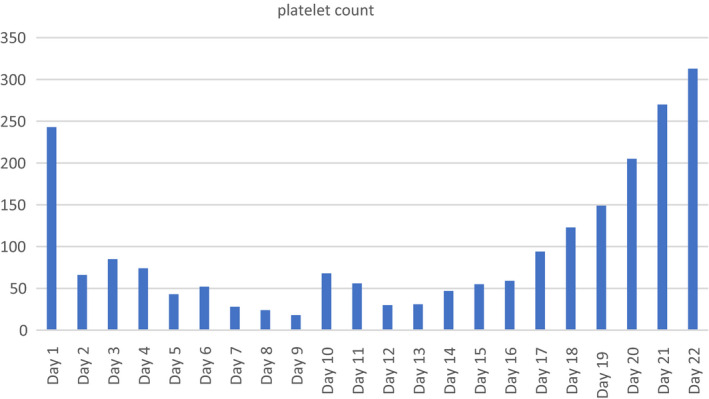
Platelet levels during course of treatment (in ×10^9^/L)

After initiation of plasma exchange, the patient's laboratory values demonstrated significant improvement: LDH level improved from 3802 to 945 IU/L within one day of plasma exchange initiation (Figure 2). Total bilirubin levels improved from 4.0 to 2.9 mg/dl within one day of plasma exchange. After the initial plasma volume exchange, her platelet count increased from 18 to 68 × 10^9^/L and continued to increase and eventually normalize thereafter, concurrent with daily plasma exchange (Figure 3). The patient also showed significant clinical improvement with declining vasopressor requirement after the second day of plasma exchange and subsequently required antihypertensives due to elevated blood pressure on the third day of plasma exchange. Peripheral smear on the fourth day of plasma exchange demonstrated a significant decrease in schistocytes per high power field. The presumption was that the rapid clinical improvement concurrent with plasma exchange was consistent with the diagnosis of TTP. However, after five consecutive plasma exchanges, the ADAMTS13 level (drawn before initiation of plasma exchange) resulted at 50.7%, decreasing the diagnostic likelihood of TTP, and plasma exchange was discontinued. The patient no longer required renal replacement therapy.

## 2 DIFFERENTIAL DIAGNOSIS

The patient initially presented with normal platelet count and with normal mentation. The patient did not have any previous issues relating to any hematologic disorders. The rapid onset of thrombocytopenia and renal failure in the context of severe COVID19 infection raised the concern for possible thrombocytopenia induced by consumptive process due to her underlying infection. In addition, COVID19‐induced thrombotic angiopathy without TTP was also considered on account of the patient's coagulopathic and inflammatory changes. The presence of a significant number of schistocytes on peripheral smear raised the concern for thrombotic thrombocytopenic purpura (TTP), despite initial presentation with normal platelet count and concurrent COVID19 infection. Due to high clinical and laboratory suspicion for TTP, plasma exchange with FFP was initiated with pending ADAMTS13 results. The patient experienced a significant improvement in her clinical status, biochemically, by laboratory values and with significant decrease in schistocytes observed per high power field. The patient's coagulation profile was not consistent with disseminated intravascular coagulation (DIC). On account of normal ADAMTS13 levels, yet clear laboratory and pathologic evidence of a thrombotic microangiopathy, our patient was diagnosed with COVID19‐induced thrombotic angiopathy, which responded briskly to plasma exchange intervention.

## 3 DISCUSSION

Patients with COVID19 infection have been found to exhibit a wide array of symptoms and complications, as published in the literature in 2020. There is a known association with COVID19 infection and a unique coagulopathy. It is postulated that this coagulopathy may be related to endothelial activation and microvascular thrombosis/hemolysis. There is evidence that viruses play an important role as a trigger in the pathogenesis of thrombotic angiopathies. The mechanism of this remains unclear; it has been suggested that direct endothelial injury by cytokine storm and immune complex mediated events along with ADAMTS13 inhibitors are implicated as underlying triggers of viral activated thrombotic microangiopathy.^1^

A study evaluating secondary thrombotic microangiopathy in COVID19‐infected patients found that patients with low ADAMTS13 levels, elevated LDH, presence of schistocytes and elevated von Willebrand factor levels were more likely to indicate severe infection and high likelihood of death.^2^

There are case reports of secondary TTP due to COVID19 infection. There is also a report by Albiol et al. of a 57‐year‐old woman diagnosed with acquired autoimmune TTP following the diagnosis of Covid‐19.^3^ Another case is reported by Hindilerden et al. of a case of TTP which was diagnosed following COVID19 infection.^4^

Martinelli et al. carried out a retrospective study on 50 admitted patients with COVID19 infection in which they evaluated various laboratory values. They found that about 2%–4% of patients had documentation of schistocytes on peripheral smears. They also found a mild decrease in ADAMTS13 level in most of the subjects (47% with 95% CI 40–55). This was the first study suggesting ADAMTS13 impairment in COVID19 infection.^5^

Our patient had a COVID‐19‐associated thrombotic microangiopathy that exhibited a dramatic response to plasma exchange. The current report adds to a growing body of literature reporting improvement with plasma exchange in severely ill patients with COVID19 infection.^6^, ^7^ It is conceivable that plasma exchange accounts for cytokine removal of inflammatory mediators in the plasma and reinstitution of immune homeostasis. It is also possible that the plasma administered contained anti‐covid antibodies that were initially popular to treat mild to moderate covid infection as covid convalescent plasma (CCP) since the dates of collection for these plasma units corresponded with CCP drives which occurred in our region, however the plasma units transfused were not tested for the presence of anti‐covid antibodies. Furthermore, most of the plasma (FFP and/or CCP) transfused was cryopoor plasma (also referred to as cryosupernatant plasma) which may have augmented the improved clinical response as they contained reduced clotting factors, in addition to the possibility of containing anti‐covid antibodies, with lower concentrations of large VWF multimers. The use of crypoor plasma in TTP has been reported as equal or preferable to general FFP^8^ although some have reported an increase in mild allergic responses.^9^ While employing plasma exchange as a therapeutic modality for COVID‐19 is sure to pose significant challenges, the emerging evidence warrants further scientific consideration. Plasma exchange (cryopoor or FFP) used on a limited scale, particularly for patients with COVID‐19 microangiopathy, may represent a useful treatment for a particularly devastating manifestation of COVID‐19.

## CONFLICT OF INTEREST

None of the authors declare any conflict of interest.

## AUTHOR CONTRIBUTIONS

NE, GR, and MHB conceived and designed the project. NH, GR, SP, JL, YH, and MHB acquired the data. NE, GR, JL, YH, SP, and MHB analyzed and interpreted the data. NE, GR, and MHB wrote the paper.

## ETHICAL APPROVAL

This work has been presented, in part, as an abstract to the American Association of Clinical Chemistry Annual Meeting and Clinical Lab Expo, September 26–30, 2021.

## CONSENT

Written Patient consent has been obtained.

## Data Availability

The data that support the findings of this study are available from the corresponding author upon reasonable request.
